# When Breathing Interferes with Cognition: Experimental Inspiratory Loading Alters Timed Up-and-Go Test in Normal Humans

**DOI:** 10.1371/journal.pone.0151625

**Published:** 2016-03-15

**Authors:** Marie-Cécile Nierat, Suela Demiri, Elise Dupuis-Lozeron, Gilles Allali, Capucine Morélot-Panzini, Thomas Similowski, Dan Adler

**Affiliations:** 1 Sorbonne Universités, UPMC Univ Paris 06, INSERM, UMRS1158 Neurophysiologie respiratoire expérimentale et clinique, Paris, France; 2 Division of Pulmonary Diseases, Geneva University Hospital and University of Geneva, Geneva, Switzerland; 3 Research Center for Statistics, Geneva School of Economics and Management, University of Geneva, Geneva, Switzerland; 4 Department of Neurology, Geneva University Hospitals and University of Geneva, Geneva, Switzerland; 5 Department of Neurology, Division of Cognitive and Motor Aging, Albert Einstein College of Medicine, Bronx, NY, United States of America; 6 AP-HP, Groupe Hospitalier Pitié-Salpêtrière Charles Foix, Service de Pneumologie et Réanimation Médicale (*Département "R3S"*), F-75013, Paris, France; University of Pécs Medical School, HUNGARY

## Abstract

Human breathing stems from automatic brainstem neural processes. It can also be operated by cortico-subcortical networks, especially when breathing becomes uncomfortable because of external or internal inspiratory loads. How the “irruption of breathing into consciousness” interacts with cognition remains unclear, but a case report in a patient with defective automatic breathing (Ondine's curse syndrome) has shown that there was a cognitive cost of breathing when the respiratory cortical networks were engaged. In a pilot study of putative breathing-cognition interactions, the present study relied on a randomized design to test the hypothesis that experimentally loaded breathing in 28 young healthy subjects would have a negative impact on cognition as tested by “timed up-and-go” test (TUG) and its imagery version (iTUG). Progressive inspiratory threshold loading resulted in slower TUG and iTUG performance. Participants consistently imagined themselves faster than they actually were. However, progressive inspiratory loading slowed iTUG more than TUG, a finding that is unexpected with regard to the known effects of dual tasking on TUG and iTUG (slower TUG but stable iTUG). Insofar as the cortical networks engaged in response to inspiratory loading are also activated during complex locomotor tasks requiring cognitive inputs, we infer that competition for cortical resources may account for the breathing-cognition interference that is evidenced here.

## Introduction

In healthy humans, normal breathing stems from automatic brainstem neural processes and does not give rise to conscious perception: it does not engage motor or sensory cortical resources. Breathing can however be operated by cortico-subcortical networks under certain circumstances, like voluntary respiratory movements or during speech [1]. Cortically driven breathing has also been described in reaction to changes in the mechanical properties of the respiratory system, namely when breathing becomes difficult [2, 3]. Externally applied inspiratory and expiratory constraints give rise to respiratory-related motor cortical activities that are associated with an augmented neural drive to breathe [2–4]. The corresponding network involves the supplementary motor cortex, with emphasis on the supplementary motor area (SMA) [5]. Similar cortical activations have been reported in patients suffering from chronic respiratory insufficiency due to respiratory muscle weakness in the contexte of amyotrophic lateral sclerosis [6] and from the obstructive sleep apnea syndrome in which upper airway abnormalities generate an "intrinsic" inspiratory load [7]. Finally, a respiratory-related cortical activity exists during resting breathing in patients with defective respiratory automatism (Ondine's curse syndrome)[8]. In one such patient, cognitive performances were better under mechanical ventilation than during to spontaneous breathing. This cognitive improvement was concomitant with a reduction in overall cortical activity, changes in brain functional connectivity (stronger connectivity between brainstem and frontal lobe during spontaneous breathing than during mechanical ventilation), and restoration of the default mode network that is associated with self-consciousness, mind-wandering, creativity and introspection [9]. This was interpreted as the result of "competition for cortical resources", in the general frame of dual tasking interferences. It could thus be postulated that respiratory diseases involving a respiratory-related motor cortical activity could be associated with executive defects through such a mechanism, and irrespective of their impact on blood oxygen and carbon dioxide. Of note, inspiratory loads give rise to respiratory discomfort and negative emotions (namely "dyspnea"). This is associated with increased metabolic activities within the limbic cortex [10] and with deactivation of the default mode network [5]. This irruption of "breathing into consciousness" could also be a called upon to explain a negative impact of dyspnea on cognitive functions, by analogy with pain [11]

**Fig 1:**
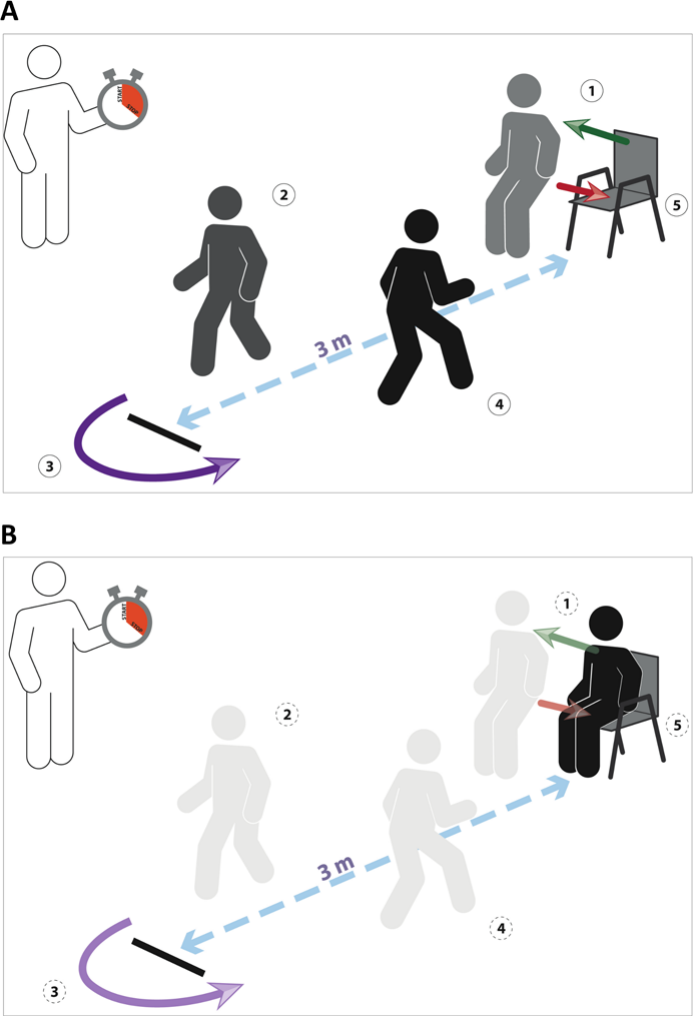
Principle of the timed-up-and-go (TUG) test (top) and of the imaginary TUG test (bottom). 1: on command, the subject rises from the armchair; 2: the subject walks 3 meters; 3: the subject turns around; 4: the subjects walks back to the chair; 5: the subjects sits back on the chair.

Similar to breathing control, gait control is considered automatic and independent from cognition. However, in elderly patients and patients suffering from certain neuropsychiatric disorders, the control of gait beccomes dependent on cognitive function and involves specific cortical regions [12]. The timed up and go task (TUG)(see Methods and Fig 1) has been largely used to assess locomotor function [13]. More recently, an imaginary version (iTUG) has been developed to evaluate the central control of locomotion [14]. Dual tasking is associated with changes in the TUG-iTUG performance [15]. In elderly individuals and in neuropsychiatric patients, TUG-iTUG changes have been assocaited with abnormalities of attention, executive function and memory [14, 16, 17]. A widening of the TUG-iTUG difference has been identified as an early biomarker of dementia [18]

Within this frame, we hypothesized that if experimental inspiratory loading, known to engage cortical resources, was associated with a cognitive cost, it would interfere with the TUG-iTUG performances. We designed the present study with the aim of testing this hypothesis by measuring TUG and iTUG in healthy volunteers submitted to a range of inspiratory loads.

## Material and Methods

### Participants and ethical approval

The study was part of a wider "breathing and posture" research program approved by the appropriate legal and ethical authority (Comité de Protection des Personnes Ile-de-France 6, La Pitié-Salpêtrière, Paris, France).

Twenty-eight healthy young subjects (16 women, 12 men; median age: 28 years, interquartile range–IQR- [24.5–38.5]) were recruited for this experiment from the campus of Université Paris 6. They reported no physical, neurological or mental disorders, and took no medication. They received detailed information about the experiment and gave their written consent to participate.

### Experiments

#### TUG and iTUG

We used the TUG test as described by Podsiadlo et al. [13] and its imagined version previously validated by Beauchet et al. [14]. In brief, the TUG test consists in asking participants to, on command ("ready, set, go"), stand up from an armchair, walk 3 meters, turn around, walk back to the chair and sit down. The participants are asked to perform the maneuver in a self-paced speed. The iTUG consists in asking the subjects to imagine themselves performing the test and verbally say "stop" out loud when they are finished. Results are given in seconds. In the present study, the participants were asked to perform the TUG and iTUG (in this order) under various breathing conditions (see below). Execution times were recorded with a stopwatch to the nearest 0.01 sec (from "go" to either complete sit down or "stop" signal). Before testing, a trained evaluator gave standardized TUG and iTUG instructions (Fig 1).

#### Breathing Conditions

Maximal inspiratory pressure was first determined by asking the subjects to produce a maximal inspiratory effort through an occluded mouthpiece [19]. The subjects wore a noseclip to prevent leaks. They were instructed to start their effort from the end of a relaxed expiration (functional residual capacity, FRC). Mouth pressure was measured through a side port of the mouthpiece, using a linear differential pressure transducer (DP 15–34, Validyne, Northridge, CA). Maximal inspiratory pressure was determined at the highest pressure sustained for 1 second during the best of three consecutive attempts.

**Fig 2:**
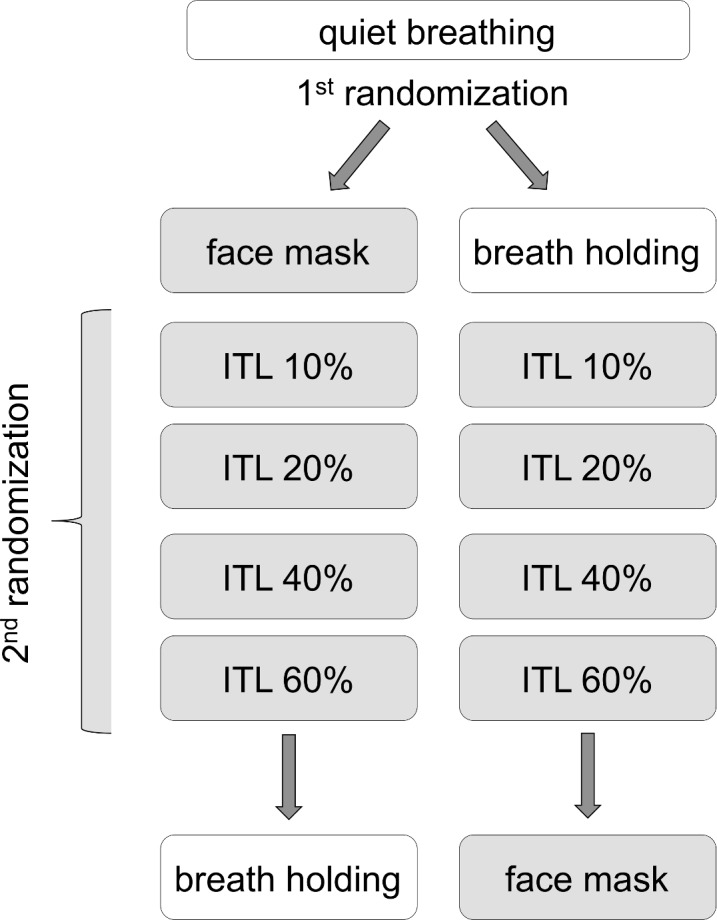
Experimental sequence and randomization. ITL: inspiratory threshold loading (in % of maximal static inspiratory pressure: 10, 20, 40, 60%).

TUG and iTUG were measured in 7 breathing conditions: a) quiet breathing; b) breathing through an oro-nasal mask (designed for non invasive ventilation) with no mechanical load attached; c) breathing against an inspiratory threshold load (Health Scan, NJ, USA, POWERBreathe, HaB, Ltd, UK) set at 10% of maximal inspiratory pressure: ITL10; d) at 20% of maximal inspiratory pressure: ITL20; e) at 40% of maximal inspiratory pressure: ITL40; f) at 60% of maximal inspiratory pressure: ITL60; g) during breath holding considered as an "infinite" load (the subjects were asked to stop breathing at the end of a relaxed expiration while signaling it to the experimenter by a nod or raising a finger; the experimenter then waited approximately 10 seconds -in order to let respiratory sensations built up- before starting the ready-set-go sequence). Conditions a was always first, followed by either "b to f then g" or by "g then b to f". The order of conditions c, d, e, and f was randomized and the subjects were blinded to the condition (Fig 2).

#### Dyspnea

Dyspnea was evaluated using a uni-dimensional visual analog scale (VAS) consisting in a 100 mm line over which the subjects were asked to displace a cursor. The scale was bounded by "no respiratory discomfort" on the left, and "intolerable respiratory discomfort" on the right. The dyspnea evaluation was performed for the quiet breathing condition and for the various levels of inspiratory threshold loading, but not for the "mask" condition neither for the "breath holding" condition. Of note, this evaluation was performed post-hoc, at the end of the complete experimental session, by asking the subjects to recall the sensation they had experienced during the different parts of the experimental sequence, in an attempt to limit the degree of unblinding of the subjects regarding the intensity of loading during the experiments.

### Statistical Analysis

Time to perform TUG and iTUG in the different breathing conditions are reported as median and interquartile ranges [IQR]. A linear mixed-effects model with a random intercept for each subject was used with the breathing condition and the type of task (TUG vs iTUG) as fixed factors. A polynomial contrast was employed to evaluate how the response time increased with the inspiratory loading. To assess whether the increase of the response time with the inspiratory loading was different for TUG or iTUG task, interaction between the two fixed factors was tested with a likelihood-ratio test. All P-values were two-sided, and statistical significance was set at a P-value of 0.05. All analyses were performed using R for Windows (version 3.2.0) 30 with the lme4, lsmeans and ggplot2 packages.

## Results

**Fig 3:**
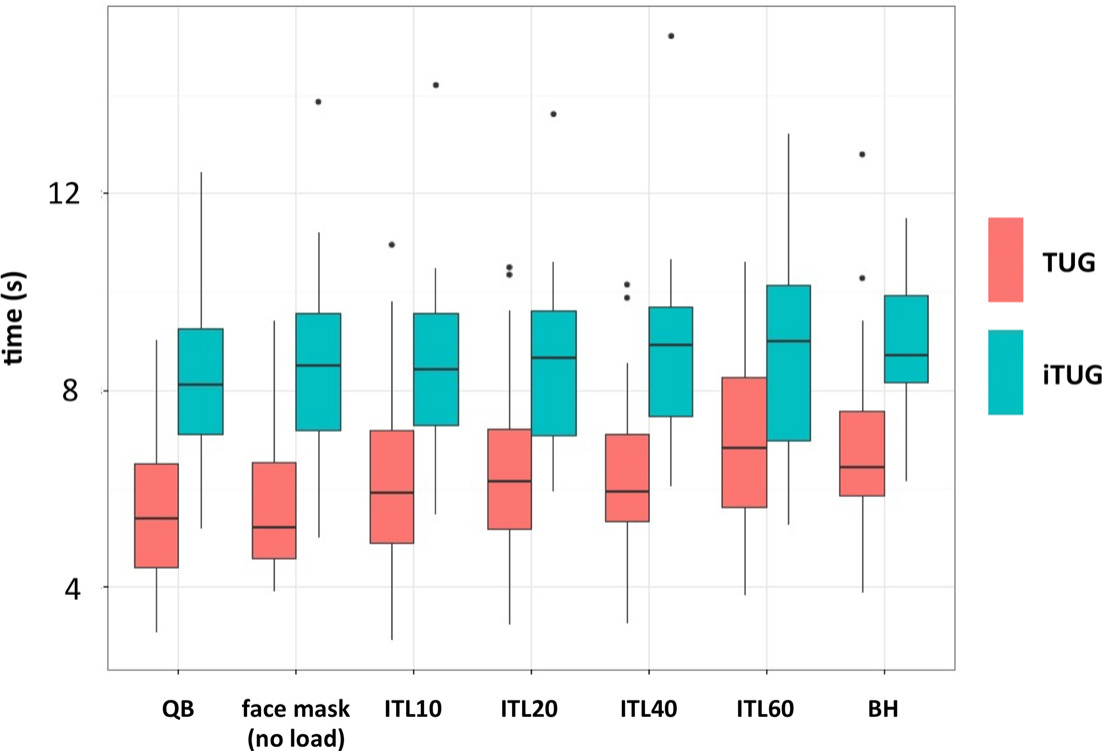
Time to perform TUG and iTUG (in seconds) under each inspiratory loading The boxes delineate the interquartile space with indication of the median within the boxes; the extremities of the whiskers correspond to the lowest datum still within 1.5 IQR of the lower quartile and the highest datum still within 1.5 IQR of the upper quartile; the individual data points represent outliers. QB: quiet breathing (no apparatus, no load); ITL: inspiratory threshold loading (in % of maximal static inspiratory pressure: 10, 20, 40, 60%); BH: breath-holding

Twenty-five participants fully completed the experiment whereas 3 did not perform the breath-holding condition (added to the protocole afterwards)(see S1 Dataset). Median TUG time during quiet breathing was 8.11 s [IQR: 7.10–9.24]. It was 9.01 s [IQR: 6.99–10.14]) during 60% ITL, and 8.70 s [IQR: 8.16–9.33] during breath-holding. Median iTUG times during quiet breathing was 5.40 s [IQR: 4.40–6.51]. It was 6.85 s [IQR: 5.36–8.26]) during 60% ITL, and 6.45 s [IQR: 5.87–7.58] during breath-holding (Fig 3). TUG and iTUG increases appeared linear insofar as only the linear component of the polynomial contrast was significant (p<0.0001): only the linear part of the contrast was therefore kept in the analyses. There was a statistically significant main effect of task (p<0.001) and a significant interaction between task and inspiratory loading (p = 0.022), suggesting that inspiratory loading has a greater effect on iTUG time than on TUG time. The median value of the observed absolute TUG-iTUG difference decreased between spontaneous breathing (2.60 s [IQR: 2.01–3.47]) and 60% ITL (2.08 s [IQR: 0.72–3.12]). It was 2.28 s [IQR: 1.39–2.62] for breath-holding. Of notice, the randomization procedure was efficient, producing 17 different sequences among the 28 subjects (10 of the sequences pertained to one subject only, 4 to 2 subjects, 2 to 3 subjects, and 1 to 4 subjects; there was no statistically significant "order" effect on the results.

The subjects did not report respiratory discomfort during quiet breathing and did not find the mask without inspiratory load meaningfully bothering. All the inspiratory loading conditions evoked dyspnea, from a median of 5 [5–6] cm (ITL 10%) to a median of 6 [6–7] cm (ITL 60%). Because of the uncertainty induced by the post hoc dyspnea evaluation (see methods), no statistical comparison was performed on this outcome.

## Discussion

This study shows that inspiratory threshold loading, known to elicit dyspnea and to engage respiratory-related cortical networks [19] interferes with the control of locomotion. Indeed, TUG and iTUG were slower under inspiratory loading than during quiet breathing. In addition, participants consistently imagined themselves being faster to perform the task than they actually were, but the TUG-iTUG difference decreased with increasing inspiratory loading (greater impact of inspiratory loading on iTUG), which is unexpected.

### Actual TUG task

This study provides the first experimental evidence that hindered breathing negatively impacts the control of locomotion. We did not record electroencephalograms to demonstrate that inspiratory loading did result in a respiratory-related cortical activity, but this is a safe assumption in view of prior results: indeed, the inspiratory loading paradigm that we used has repeatedly been shown to elicit a consistent pattern of EEG responses in normal subjects [2–4, 20, 21]. It can thus be postulated that, in our subjects, having to breathe against an inspiratory load engaged cortical resources that competed with those involved by the TUG task. This fuels the concept of breathing-cognition interferences, in line with observations made in a patient suffering from central congenital alveolar hypoventilation (see introduction). A similar competition for cortical resources has been reported for locomotion during dual tasking paradigms [22–26]. Of note, dual-tasking does deteriorate TUG performance [15]. Functionally, inspiratory loading activates a cortico-subcortical network comprising the anterior cingulate gyrus, the insula, and premotor and motor cortices [5], all areas that also engaged in complex locomotor tasks [27], making "cortical competition" a reasonable hypothesis. In this view, patients with chronic obstructive pulmonary disease (COPD) patients exhibit gait abnormalities that worsen with disease severity [28]), and patients suffering from sleep disordered breathing exhibit gait abnormalities that have been associated with attention and executive function [29, 30]. Yet recent data demonstrate that patients with severe forms of the obstructive apnea syndrome exhibit a inspiratory loading like respiratory-related cortical activity [7]. From our present observations, respiratory dual tasking could be called upon to explain gait abnormalities associated with obstructive sleep apneas, and possibly other respiratory diseases.

### Imaginary TUG and TUG-iTUG difference

In line with published data, we observed iTUG times shorter than TUG times, with a TUG-iTUG difference that was within the expected range [16, 17, 31, 32]. Both TUG and iTUG times increased with inspiratory loading and became maximal during breath-holding, but iTUG increased more than TUG, which resulted in a decreased TUG-iTUG difference. This contrasts with previously reported results showing that dual tasking increases TUG and iTUG but widens the TUG-iTUG difference [15], as do aging [14] and several neuropsychiatric conditions [15–17, 31, 32]. The age-related increased in the TUG-iTUG difference has been interpreted as the consequence of a preserved motor imagery in situations where the actual execution of a complex task requires more cortical activation to compensate for the age-related decline in performance [33, 34].

The mechanisms leading to a decreased TUG-iTUG difference in our subjects are not obvious and will have to be studied specifically. One possible explanation could be an inspiratory loading related change in the sense of respiratory agency, namely the notion that breathing results from self-generated mechanisms and not from the intervention of external agents (similar notion regarding locomotion in [35]). In normal subjects, the mere ungating of respiratory afferents by a visual signal synchronized with breathing is sufficient to modify respiratory agency [36]. This effect could also result from inspiratory loading, making motor imagery more congruent with actual motor task and therefore narrowing the TUG-iTUG difference. Alternatively, the threatening sensations induced by inspiratory loading could invade consciousness and make it difficult for the subjects to adequately imagine the TUG task or concentrate on it. This raises the question of the role of dyspnea "per se" in the cognitive decline that is associated with chronic respiratory diseases such as chronic obstructive pulmonary disease (COPD), a role that has seemingly not been studied specifically [37].

### Strengths, weaknesses and perspectives

We acknowledge that the study population (n = 28) is limited in size. However, we found a statistically significant dose-response relationship between the intensity of the respiratory load and time to perform TUG and iTUG, an observation that is rendered all the more meaningful by the randomised design. This suggests a causative relationship and indicates that subject-dependent factors did not play a major role. However, this is a "proof of concept" study that describes a phenomenon without explaining it. In particular, the role of the inspiratory load itself and the role of the associated dyspnea cannot be disentangled from our results. This is mostly because the evaluation of dyspnea that we performed was far from optimal. Indeed, we did not ask the subjects to rate their respiratory sensations after each breathing condition but only at the end of the experimental sequence. This was intended to minimize the realization of the magnitude of the inspiratory loads and limit interferences with the randomized design of the study, but this probably resulted in the successive sensations being blurred by the recall process. This could explain why, surprisingly, the dyspnea ratings for ITL10 and ITL60 were nor very different. Specifically desgined studies will therefore be necessary. Also, we are aware that breath-holding and inspiratory threshold loading probably involve very different brain mechanisms. Breath-holding however appeared interesting to add to the experimental sequence as a "maximal control", during which the active task of inhibiting breathing would correspond to an extreme form of cortical efferent output to the respiratory system, and during which the brain would be inundated with respiratory sensations.

The above limitations confer a preliminary nature to our data. Our results will require corroboration, and in particular it will be necessary to study the impact of respiratory constraints on other types of cognitive tasks. Here we chose the TUG test in part because an interaction of breathing with locomotion would be clinically relevant to chronic respiratory disorders [28, 29] and because the TUG test has been shown to be sensitive to dual tasking. It is however an integrative procedure exploring motor and cognitive processes hence the possibility of biases. We however reasoned that it would be very difficult to experimentally compromise the motor component of the TUG test in normal subjects, and therefore that changes in response to inspiratory loading would more probably reflect a cognitive impact. Our results will also require explanation (cortical competition? motor, sensory, or both?). This study is therefore preliminary and fragmentary, but we believe that it could represent the starting point of a completely new research area devoted to unravel interactions between breathing and cognition.

## Supporting Information

S1 Dataset Raw TUG-iTUG data in the 28 subjects. (CSV)
